# Sex and Age: 2 Predictors of Cardiovascular Events With COVID-19 Infection∗

**DOI:** 10.1016/j.jacadv.2023.100325

**Published:** 2023-05-10

**Authors:** Gina Lundberg, Olga Toleva, Alexis Cutchins

**Affiliations:** Division of Cardiology, Department of Medicine, Emory University School of Medicine, Atlanta, Georgia, USA

**Keywords:** cardiovascular events, cardiovascular risk factors, COVID-19, sex-specific outcomes

**Figure:**
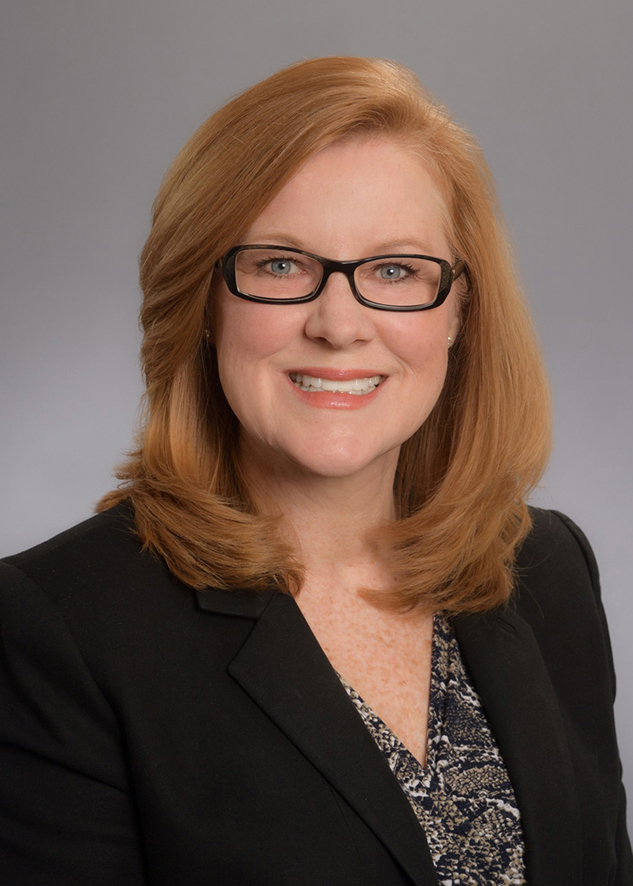

The pandemic from COVID-19 from SARS-CoV-2 infection has been with us for 3 years in North America. Many healthcare workers have experienced burnout and exhaustion as a result. Almost one-half of all public health workers have quit their jobs or retired in the last 5 years, many due to COVID-19.^1^ Many healthcare workers are experiencing “COVID fatigue” at this point. Yet if we are to truly put COVID-19 in the rear-view mirror, we must continue to study COVID-19 infections to determine best practices for preventing and treating infections to reduce cardiovascular (CV) events.

The paper Behrouzi et al^2^ in this issue of *JACC: Advances* reveals data from a massive data base in a country with socialized medicine across the first 3 waves of the pandemic. This study is a seminal example of a well-conducted and very large population-based epidemiological study using strong captured administrative data. Although such data may lack clinical risk adjusters such as oxygen saturation, symptomatology, respiratory rate, it is still highly valuable for answering the research question. As highlighted rightfully by the authors, they included almost all patients in Ontario with laboratory confirmed COVID-19 infection, had access to gold standard test results, and focused analyses on highly identifiable outcomes of interest that are nonsusceptible to ascertainment biases. Statistical analysis was extremely careful to account for known confounding factors.

We commend the authors’ work evaluating sex and age differences in CV events, including all-cause hospitalizations and all-cause deaths, in the acute setting of SARS-CoV-2 infection. Sex differences in severity and outcomes in COVID-19 infections have been evident since the first outbreak in North America.^3^ Male sex has been independently associated with hospitalization, need for intensive care, ventilatory support, and death.^4^ COVID-19 severity and mortality have also varied between sexes based on age and other chronic conditions. CV risk factors including hypertension, diabetes, heart failure, coronary artery disease as well as chronic lung and kidney disease have been associated with worse outcomes with COVID-19 as well. Multiple studies have shown these findings in Europe, Japan, and globally.^5^

The main finding in this study is that males were consistently, through all age strata, at higher risk of adverse outcomes including CV events. The authors demonstrate increased sex-specific risk for males over females during all 3 waves of the pandemic, noting this was not due to disparities in testing. In fact, more females than males were tested during the first wave. Across all 3 waves, males experienced more severe disease and fatality than females after adjusting for comorbidities and socioeconomic factors.

Males remain more adversely affected overall in the acute phase of infection for all populations except one (females ages 18-45 years old during the second and third waves of the pandemic). Interestingly, the population most affected by long COVID and lingering symptoms is younger females, although males are affected at a lower extent.^6^ Subramanian et al^6^ describe an average age of 43.8 years old and 55.3% females in their study of long COVID. This increase in hospitalizations in younger females during the second and third waves is potentially the onset of symptoms of long COVID that may warrant hospitalization. The time delay from the positive COVID-19 test and the hospitalization episode would be key to answering this question but is lacking in the analysis. While a diagnosis of long COVID cannot be entertained until >4 weeks after infection, patients can develop the start of these prolonged symptoms immediately after infection and may even be hospitalized initially during that 30-day post infection period.

When comparing hospitalizations and admissions for CV events across the 3 waves, it is interesting to see a consistent difference between males and females. There is a substantial drop in hospitalizations for both males and females between the first wave and the second and third waves across the 46 to 85-year-old age groups, which is shown in the Central Illustration of Behrouzi et al^2^. The youngest and the oldest patients appear to be outliers. This may result from the vaccine being less effect in those over 80 years of age and more effective in the younger group and underscore the importance of age and pre-existing conditions in relation to the severity of the infection.^7^^,^^8^ The findings support the idea that the older age groups (>80 years) may not have had as robust of a response to the vaccine.^7^^,^^9^ Of note, one study looking at sex differences in efficacy of the COVID-19 vaccine showed higher efficacy in males over females which may speculate that the sex-specific findings of Behrouzi et al^2^ may have been even more significant had a vaccine not been developed.^9^

Importantly, this study confirms that across the first 3 waves of COVID-19 infections, the most severe outcomes were seen among older patients, more male patients, patients with higher numbers of comorbidities and among frail patients. Males experienced higher rates of severe outcomes compared with females with severe outcomes increasing with age for both sexes. The incidence of CV hospitalization across the first 3 waves revealed males ages 46 to 65 years were at the highest risk, 3-fold higher than seen in females, with the most common CV hospitalizations due to arrhythmias, heart failure, stroke, and acute myocardial infarction.

Finally, the most important and reassuring finding to us is that all-cause mortality decreased substantially by the third wave, and the male dominance in death profile tended to disappear. In each new wave, sex disparities reduced for all outcomes except for CV hospitalization. In the current era of herd immunity and multiple vaccines, it is impossible to generalize the study findings and predict the future. Nevertheless, this study showed the value of vaccination to reduce adverse outcomes and bringing males’ outcomes similar to females for COVID-related outcomes. In this stage of COVID fatigue and vaccine resistance, non-vaccinated individuals of all ages should be informed that males have a poorer prognosis and higher risk with COVID-19 infection than females. Physicians and healthcare workers should remain active and engaged in educating patients on the life-saving value of vaccines.

The actionable results from this study are to encourage men of all ages, aging women, frail patients, and those with recent hospitalizations, hypertension, diabetes, heart failure, atrial fibrillation, chronic kidney disease, cancer, and lung disease to remain current with vaccinations and follow preventive measures to avoid COVID-19 infection. And these patients should seek medical care at the first signs of arrhythmias, heart failure, stroke, or heart attack. Despite tremendous public health efforts, COVID-19 is still virulent and remains a significant health threat with CV events that still lead to hospitalization, morbidity, and even mortality.

## Funding support and author disclosures

The authors have reported that they have no relationships relevant to the contents of this paper to disclose.
